# A Toxicologic Review of Quantum Dots: Toxicity Depends on Physicochemical and Environmental Factors

**DOI:** 10.1289/ehp.8284

**Published:** 2005-09-20

**Authors:** Ron Hardman

**Affiliations:** Nicholas School of the Environment and Earth Sciences, Duke University, Durham, North Carolina, USA

**Keywords:** environment, human health, nanomaterials, nanosized particles, nanotechnology, nanotoxicology, quantum dots, toxicology

## Abstract

As a growing applied science, nanotechnology has considerable global socioeconomic value, and the benefits afforded by nanoscale materials and processes are expected to have significant impacts on almost all industries and all areas of society. A diverse array of engineered nanoscale products and processes have emerged [e.g., carbon nanotubes, fullerene derivatives, and quantum dots (QDs)], with widespread applications in fields such as medicine, plastics, energy, electronics, and aerospace. With the nanotechnology economy estimated to be valued at $1 trillion by 2012, the prevalence of these materials in society will be increasing, as will the likelihood of exposures. Importantly, the vastness and novelty of the nanotechnology frontier leave many areas unexplored, or underexplored, such as the potential adverse human health effects resulting from exposure to novel nanomaterials. It is within this context that the need for understanding the potentially harmful side effects of these materials becomes clear. The reviewed literature suggests several key points: Not all QDs are alike; engineered QDs cannot be considered a uniform group of substances. QD absorption, distribution, metabolism, excretion, and toxicity depend on multiple factors derived from both inherent physicochemical properties and environmental conditions; QD size, charge, concentration, outer coating bioactivity (capping material and functional groups), and oxidative, photolytic, and mechanical stability have each been implicated as determining factors in QD toxicity. Although they offer potentially invaluable societal benefits such as drug targeting and *in vivo* biomedical imaging, QDs may also pose risks to human health and the environment under certain conditions.

In 1959 Richard Feynman’s seminal talk on nanotechnology, “There’s Plenty of Room at the Bottom,” presented what was theoretically possible by manipulating matter at the atomic and molecular scales. Today, nanotechnology is an applied science, a rapidly growing industry generating a diverse array of nanoscale materials and processes (e.g., carbon nanotubes, fullerene derivatives, quantum dots). Manipulation of materials and processes on a nanometer scale is opening a world of creative possibilities, and the benefits afforded by nanoscale technologies are expected to have substantial impacts on almost all industries and areas of society (e.g., medicine, plastics, energy, electronics, aerospace). It is such creative potential that renders nanotechnology of significant social and economic value. With approximately $8.6 billion invested in nanotechnology research and development worldwide in 2004 (Nordan et al. 2004), and a projected nanotechnology economy valued at $1 trillion by 2012 (Service 2004), the prevalence of these materials in society is ensured, and human exposures, as well as those of wildlife, are likely to increase. Currently, nanotechnology products are sold by more than 200 companies globally; some are widely used in commercially available products (e.g., electronic, cosmetic) (Hood 2004; National Science Foundation 2004). For perspective on the size of nanoscale products, consider that 2 g of 100 nm-diameter nanoparticles contains enough material to provide every human worldwide with 300,000 particles each.

The nascent nature of the nanotechnology industry, however, leaves many areas unexplored, or underexplored, such as the potential adverse effects of engineered nanomaterials on human health and the environment. Currently, the paucity of toxicologic information and lack of standardized testing protocols make assessment of the adverse effects of engineered nanosized materials on biologic systems difficult (National Toxicology Program 2005; U.S. Environmental Protection Agency 2003). The growing prevalence of nanomaterials in society, in conjunction with their unique physicochemical properties and the risk of unwanted/unanticipated exposures, renders them of potential concern to human health and the environment. It is within this context that the need for understanding the potentially harmful side effects of these materials is becoming clear (Colvin 2003; Oberdörster et al. 2005).

Reviewed here are novel nanomaterials commonly referred to as quantum dots (QDs). Although they offer potentially invaluable societal benefits such as drug targeting and *in vivo* biomedical imaging (Alivisatos 2004; Gao et al. 2004; Michalet et al. 2005; Roco 2003), they may also, as the reviewed literature suggests, pose risks to human health and the environment under certain conditions. Current literature reveals that assessing QD exposure routes and potential toxicity is not a simple matter; not all QDs are alike, and toxicity depends on multiple physicochemical as well as environmental factors.

## Applications of Quantum Dots

Quantum dots are semiconductor nanocrystals (~ 2–100 nm) with unique optical and electrical properties (Bruchez et al. 1998; Dabbousi et al. 1997) currently applied in biomedical imaging and electronics industries. One of the more valuable properties of QDs is their fluorescence spectrum, which renders them optimal fluorophores for biomedical imaging (Alivisatos 2004; Chan et al. 2002). For instance, fluorescent QDs can be conjugated with bioactive moieties (e.g., antibodies, receptor ligands) to target specific biologic events and cellular structures, such as labeling neoplastic cells (Gao et al. 2004; Wu et al. 2003), peroxisomes (Colton et al. 2004), DNA (Dubertret et al. 2002), and cell membrane receptors (Beaurepaire et al. 2004; Lidke et al. 2004). Bioconjugated QDs are also being explored as tools for site-specific gene and drug delivery (Rudge et al. 2000; Scherer et al. 2001; Yu and Chow 2005) and are among the most promising candidates for a variety of information and visual technologies; they are currently used for the creation of advance flat-panel LED (light-emitting diode) displays and may be employed for ultrahigh-density data storage and quantum information processing (Wu et al. 2004).

## Quantum Dot Physicochemical Properties

**Figure 1:**
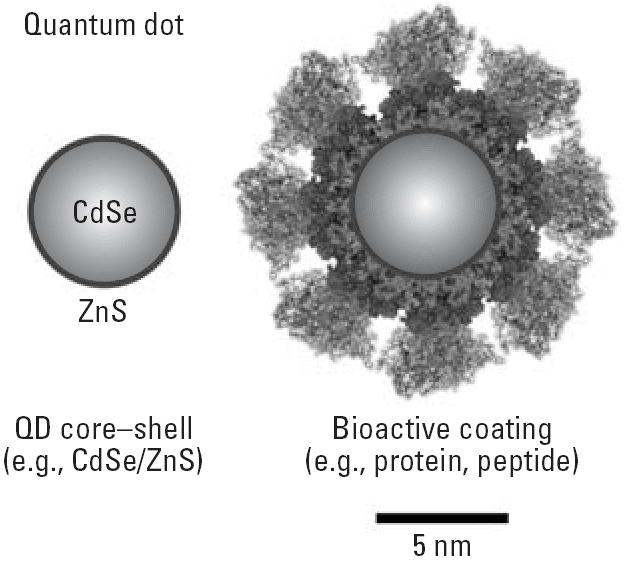
QDs consist of a metalloid core and a cap/shell that shields the core and renders the QD bioavailable. The further addition of biocompatible coatings or functional groups can give the QD a desired bioactivity.

Understanding the potential toxicity of QDs requires a fundamental grasp of QD physicochemical properties. Although naturally occurring biogenic and anthropogenic nanosized particles abound in nature, engineered QDs differ because of unique physicochemical properties that result from a combination of their crystalline metalloid core structure/composition and quantum-size confinement, which occurs when metal and semiconductor particles (QD cores) are smaller than their Bohr radii (~ 1–5 nm). Structurally, QDs consist of a metalloid crystalline core and a “cap” or “shell” that shields the core and renders the QD bioavailable (Figure 1). QD cores consist of a variety of metal complexes such as semiconductors, noble metals, and magnetic transition metals. For instance, group III–V series QDs are composed of indium phosphate (InP), indium arsenate (InAs), gallium arsenate (GaAs) and gallium nitride (GaN) metalloid cores, and group II–IV series QDs, of zinc sulfide (ZnS), zinc–selenium (ZnSe), cadmium–selenium (CdSe), and cadmium–tellurium (CdTe) cores (Dabbousi et al. 1997; Hines and Guyot-Sionnest 1996). Synthetic routes to newer heavier structures (e.g., CdTe/CdSe, CdSe/ZnTe) and hybrids composed of lead–selenium (PbSe) have also been established (Kim et al. 2003).

Further assignation of biocompatible coatings or functional groups to the QD core–shell can give QDs a desired bioactivity. Newly synthesized QDs are inherently hydrophobic in nature and not biologically useful, given a hydrophobic cap formed on the metalloid core during their synthesis in organic solvents. To render them biologically compatible/active, newly synthesized QDs are “functionalized,” or given secondary coatings, which improves water solubility, QD core durability, and suspension characteristics and assigns a desired bioactivity. For example, QD cores can be coated with hydrophilic polyethylene glycol (PEG) groups to render QDs biocompatible and can be further conjugated with bioactive moieties to target specific biologic events or cellular structural features (described above). Hence, bonding various molecular entities to the QD core functionalizes QDs for specific diagnostic or therapeutic purposes. Functionalization may be achieved via electrostatic interactions, adsorption, multivalent chelation, or covalent bonding, important physicochemical features when considering QD durability/stability and *in vivo* reactivity. In the literature, QD physicochemical characteristics are typically referred to as “core–shell-conjugate” or vice versa. CdSe/ZnS, for example, would refer to a QD with a CdSe core and ZnS shell, and a CdSe/ZnS QD conjugated with sheep serum albumin (SSA) would be referred to as CdSe/ZnS–SSA. Controlling the physicochemical properties during synthesis, which can be done with high precision, allows tailoring QDs for specific functions/uses.

Herein lies both their strength and weakness: QDs can be given highly specific bioactivities by tailoring their coatings, for example, for diagnostic (e.g., molecular imaging) and therapeutic (e.g., drug delivery) purposes. Their potential weakness is in the very coating that makes them valuable: Compromise of the coating can reveal the metalloid core, which may be toxic either as a composite core (e.g., CdTe) or upon dissolution of the QD core to constituent metals (e.g., Cd). Degradation of the QD coating may also result in reaction of the QD in undesirable/unanticipated ways *in vivo*. Further, some QD coating materials have themselves been found to be cytotoxic, such as mercaptoacetic acid (MAA; discussed further below). From this, it can be seen that QD physicochemical properties are fundamental to understanding QD toxicity; it is the stability of QD core-coating bioactive complexes that may render QDs potentially harmful, and because QDs have been found to degrade under photolytic and oxidative conditions, QD stability likely will figure significantly in commercialization of QD products.

## Quantum Dot Toxicity

Discussion of QD toxicity can be somewhat confusing because of the diversity QDs being synthesized. To make a review of this topic simpler, it should be made clear that not all QDs are alike. Each individual type of QD possesses its own unique physicochemical properties, which in turn determines its potential toxicity or lack thereof. In general, there are discrepancies in the current literature regarding the toxicity of QDs that can be attributed to several factors: the lack of toxicology-based studies, the variety of QD dosage/exposure concentrations reported in the literature, and the widely varying physicochemical properties of individual QDs. Studies specifically designed for toxicologic assessment (e.g., dose, duration, frequency of exposure, mechanisms of action) are few. Many of the studies from which QD toxicity information is derived and that have been cited in reference to QD toxicity were performed by nanotechnology researchers rather than toxicologists or health scientists. Most of the current studies reviewed here were designed to ask questions concerning the physicochemical properties of novel QD products such as fluorescence, detectability, stability, and cell labeling efficacy, not QD toxicity per se.

Importantly, and a potential source of confusion in assessing QD toxicity, QD toxicity depends on multiple factors derived from both individual QD physicochemical properties and environmental conditions: QD size, charge, concentration, outer coating bioactivity (capping material, functional groups), and oxidative, photolytic, and mechanical stability have each been shown to be determining factors in QD toxicity. For example, some QDs have been found to be cytotoxic only after oxidative and/or photolytic degradation of their core coatings. Last, because QD dosage/exposure concentrations reported in the literature vary in their units of measurement (e.g., milligrams per milliliter, molarity, milligrams per kilogram body weight, number of QDs per cell), correlating dosage across current studies is challenging. Following is a review of *in vitro* and *in vivo* studies that describe the characteristics of QDs that may render them potentially toxic to vertebrate systems.

### Routes of exposure.

Although the potential adverse effects of nanomaterials on the environment and human health have recently been addressed by research initiatives organized under the National Science Foundation and the U.S. Environmental Protection Agency, no factual information is currently available regarding routes of QD exposure. QD stability, aerosolization, half-lives, and how they partition into environmental media are currently poorly understood. However, consideration of exposure routes may be extrapolated from what is known regarding materials of similar size and physicochemical properties.

Potential routes of QD exposure are environmental, workplace, and therapeutic or diagnostic administration. Workplace exposures (e.g., engineers, researchers, clinicians) may result from inhalation, dermal contact, or ingestion. For inhalation routes, an extensive body of toxicologic research exists on other nanoscale particles (e.g., asbestos, ultrafine particles) that may provide a foundation from which to approach QD inhalation studies. QDs vary in size, ranging from approximately 2.5 nm up to 100 nm, depending on coating thickness, and vary in their sites of deposition in pulmonary tissues once aerosolized. For instance, QDs < 2.5 nm may reach the deep lung and interact with the alveolar epithelium, whereas larger aerosolized QDs deposit in bronchial spaces. However, under what conditions QDs aerosolize and whether they form aggregates in ambient air are not known (a salient review on nanomaterials and inhalation exposures is given by Oberdorster et al. 2005). Inhalation exposures may pose potential risks given that QDs have been shown to be incorporated via endocytosis by a variety of cell types and may reside in cells for weeks to months. What risks exposures via dermal absorption and accidental ingestion may pose is currently unknown.

What will likely be a significant concern as a route of exposure, given the social and economic value of therapeutic/diagnostic QD products, are exposures resulting from QD administration to humans for medicinal purposes. These types of exposures are at present theoretical, as QD products are not currently approved for therapeutic/diagnostic purposes; however, the potential for undesired/unanticipated effects resulting from medicinal/diagnostic administration of these materials likely will figure prominently in the development of medically based QD products. Their potential toxicity via administrative routes of exposure is highly dependent on a suite of variable and poorly understood factors: QD toxico- and pharmacokinetics, toxico- and pharmacodynamics, and *in vivo* stability. It may be that once QD kinetics and dynamics are characterized, the risks posed by these exposures may be mitigated through quality control mechanisms (e.g., consistency and reliability in volume production), as they currently are with pharmaceuticals.

Exposures through environmental media (contamination) are a potential route of concern primarily because of QD metalloid core compositions, and to some extent because of QD core coatings. Many QD core metals (e.g., Cd, Pb, Se) are known to be toxic to vertebrate systems at relatively low concentrations (parts per million); however, understanding the risks posed by QDs in the environment will prove complex, as toxicity varies widely with the chemical state of the metals, and environmental transformation/degradation and partitioning will determine the level of the human health hazard. Currently, nothing is known regarding the stability of QDs in the environment, product lifetimes, or how these materials partition into environmental media. Introduction of QDs into environmental media may occur via waste streams from industries that synthesize or use QDs and via clinical and research settings. Consequently, disposal of QD materials and the risks of leakage and spilling during manufacturing and transport are potential sources of concern. Environmental exposures are a significant source for several reasons: *a*) the environmental concentration of anthropogenic substances increases in direct proportion to their use in society, and QDs, given their wide range of applications, may see substantial production volumes; *b*) the half-lives of these materials may be quite long (months to possibly years); and *c*) environmental exposure will depend on where these materials partition (e.g., air, water, soil types). Because of the diversity of physicochemical properties among varied types of QDs, it is likely that elucidating environmental partitioning will be difficult. These are important considerations given that degradation of these materials in environmental media, in the event they reach environmental compartments, will undoubtedly occur, and their rates of decay are likely to be highly variable, depending on both QD physicochemical characteristics and the environmental media in which they partition. As mentioned, certain types of QDs have been shown to degrade under photolytic and oxidation conditions (discussed further below).

Although little information currently exists regarding routes of QD exposure, all routes described are of potential concern given QDs have been shown to be incorporated into a variety of cell types via endocytotic mechanisms. Current research also suggests that there may be a risk of bioaccumulation of these materials (e.g., metals) in organs and tissues, as QDs have been shown to reside in cells for weeks to months and potentially may present problems with body burdens. Common to all routes of exposure is the issue of QD stability. Virtually nothing is known about QD metabolism in vertebrate organisms or their routes of excretion. Although QDs have been shown to degrade under photolytic and oxidative conditions, degradation products have not been identified/defined *in vivo* except for the release of component core metals such as Cd and Se. Finally, in considering routes of exposure, it is important to remember that not all QDs are alike; each individual QD type possesses its own unique physicochemical properties that will dictate its likely route of exposure.

### QD cytotoxicity.

*In vitro* studies suggest certain QD types may be cytotoxic. Lovric et al. (2005) found that CdTe QDs coated with mercaptopropionic acid (MPA) and cysteamine were cytotoxic to rat pheochromocytoma cell (PC12) cultures at concentrations of 10 μg/mL. Uncoated CdTe QDs were cytotoxic at 1 μg/mL. Cell death was characterized as chromatin condensation and membrane blebbing, symptomatic of apoptosis. Cytotoxicity was more pronounced with smaller positively charged QDs (2.2 ± 0.1 nm) than with larger equally charged QDs (5.2 ± 0.1 nm) at equal concentrations (cytotoxicity determined by MTT [3-(4,5-dimethylthiazol-2-yl)-2,5-diphenyltetrazolium bromide] assay. QD size was also observed to affect subcellular distribution, with smaller cationic QDs localizing to the nuclear compartment and larger cationic QDs localizing to the cytosol. The mechanisms involved in cell death were not known but were considered to be due to the presence of free Cd (QD core degradation), free radical formation, or interaction of QDs with intracellular components leading to loss of function. The effect of QD-induced reactive oxygen species on cell death was assessed with *N*-acetylcysteine (NAC; a known inhibitor of Cd toxicity), bovine serum albumin (BSA), and Trolox (a water-soluble vitamin E). Both NAC and BSA but not Trolox significantly reduced CdTe QD toxicity, suggesting that QD-induced toxicity may be partially induced by Cd. A similar study by Hoshino et al. (2004a) found that treatment with the QD capping material mercaptoundecanoic acid (MUA) alone (without QD) for 12 hr caused severe cytotoxicity in murine T-cell lymphoma (EL-4) cells at 100 μg/mL. Treatment with cysteamine alone proved weakly genotoxic at 100 μg/mL (12 hr). Hence, in the Hoshino et al. (2004a) study, cytotoxicity was attributed to QD capping material rather than the core metalloid complex itself. It is, however, unlikely that the toxicity observed by Lovric et al. (2005) can be solely attributed to the QD coatings (MPA and cysteamine), as both size and charge and the effects of NAC and BSA suggest otherwise. Briefly, CdTe QD–induced cytotoxicity in the Lovric et al. study was shown to be partly dependent on QD size and may be due to QD coating, intracellular reactions of the surface coatings, or intracellular degradation of QDs to metalloid ions. QD-induced cytotoxicity was also observed by Shiohara et al. (2004): MUA-coated CdSe/ZnS QDs were observed to be cytotoxic to HeLa cells and primary human hepatocytes at concentrations of 100 μg/mL (MTT assay).

Several *in vitro* and *in vivo* studies have been cited in the literature as demonstrating a lack of evidence for QD-induced cytotoxicity (Ballou et al. 2004; Dubertret et al. 2002; Hoshino et al. 2004a; Jaiswal et al. 2003; Larson et al. 2003; Voura et al. 2004). However, a few of the above studies do suggest that QDs can affect cell growth and viability. QD micelles, CdSe/ZnS QDs in a hydrophobic core of *n*-polyethyleneglycol phosphatidylethanolamine (PEG–PE) and phosphatydilcholine, resulted in cell abnormalities (viability, motility) when injected into *Xenopus* blastomeres at concentrations of 5 × 10^9^ QDs/cell (~ 0.23 pmol/cell), whereas cells injected with 2 × 10^9^ QDs/cell exhibited a normal phenotype and were said to be statistically similar to uninjected embryos (Dubertret et al. 2002). Hence, QD cytotoxicity was dose dependent. Hoshino et al. (2004a) also found QD-induced cytotoxicity to be dose dependent. EL-4 cells incubated (10^6^ cells/well) with concentrations of 0.1, 0.2, and 0.4 mg/mL of CdSe/ZnS–SSA QDs exhibited a dose–response relationship (24 hr). Cell viability decreased at QD concentrations above 0.1 mg/mL, and almost all cells incubated with 0.4 mg/mL were nonviable beyond 6 hr. Interestingly, approximately 10% of EL-4 cells retained QDs after 10 days of culture. The fluorescence intensity (QDs) of cells gradually decreased and was highly concentrated in endosomes, suggesting intracellular degradation of QDs. Although cytotoxicity was observed at 0.1 mg/mL *in vitro*, EL-4 cells incubated in 0.1 mg/mL SSA-conjugated QDs, and subsequently injected into nude mice (iv), were not observed to be toxic *in vivo*. In a subsequent study, Hoshino et al. (2004b) observed reversible DNA damage in WTK1 cells (comet assay). DNA damage was noted at 2 hr of treatment with 2 μM QD–COOH (carboxylic acid), but after 12 hr QD-induced DNA damage was efficiently repaired.

QD-induced cytotoxicity was not observed in several *in vivo* and *in vitro* studies. In an *in vivo* study employing mice, Ballou et al. (2004) injected (iv) amphiphilic polyacrylic acid polymer-coated QDs (amp-QDs), and amp-QDs conjugated to PEG-amine groups (mPEG–QDs), at QD concentrations of 20 pmol QD/g animal weight. Necropsy showed no signs of necrosis at the sites of tissue deposition, and injected mice were viable for 133 days until the time of necropsy. No obvious sign of QD breakdown *in vivo* was detected by electron microscopy (*in vivo* QD stability was presumed to be due to the amphiphilic polymer coating). In another *in vivo* study, Larson et al. (2003) observed no noticeable ill effects in mice injected (iv) with 20 nM and 1 μM solutions of CdSe/ZnS QDs (“ill effects” was not defined). Voura et al. (2004), treating B16F10 melanoma cells with dihydroxylipoic acid (DHLA)–capped CdSe/ZnS QDs (5 μL/mL), noted no detectable difference in growth between QD-treated and untreated cells. Similarly, HeLa and *Dictyostelium discoideum* cells treated with 400–600 nM concentrations of CdSe/ZnS QDs capped with DHLA were observed to remain stably labeled for more than a week with no detectable effects on cell morphology or physiology (Jaiswal et al. 2003). Hanaki et al. (2003), exposing Vero cells to 0.24 mg/mL (2-hr exposure, cells washed and reincubated) CdSe/ZnS QDs capped with MUA and coated with SSA, found no effect of QDs on cell viability (MTT assay). Although it was noted that Vero cells without MUA–QD granules dominated the population during successive cell divisions, the authors stated they could not eliminate the possibility that MUA-capped QDs affect the cell viability when MUA-capped QDs are distributed in the cytosol, because they had not investigated it. Last, Chen and Gerion (2004), using CdSe/ZnS QDs conjugated with the viral SV40 nuclear localization signal peptide, observed no cytotoxicity in HeLa cells transfected with the peptide-coated QDs. The authors observed that QD concentrations of 100 pmol/10^6^ cells (~ 100 nM QD concentration) had minimal impact on cell survival (measured by colonigenic assay).

### Photolysis and oxidation: QD stability.

Possibly the most important aspect of QD toxicity is their stability, both *in vivo* and during synthesis and storage. Several studies suggest QD cytotoxicity to be due to photolysis or oxidation. Under oxidative and photolytic conditions, QD core–shell coatings have been found to be labile, degrading and thus exposing potentially toxic “capping” material or intact core metalloid complexes or resulting in dissolution of the core complex to QD core metal components (e.g., Cd, Se). Primary rat hepatocytes exposed to 62.5 μg/mL MAA–CdSe QDs exhibited cell death, attributed to photo lysis and oxidation of the QD coating. The hepatotoxicity of MAA–tri-*n*-octylphosphine oxide (MAA–TOPO)-capped CdSe QDs was found to be dependent on QD processing conditions and QD dose (Derfus 2004). If MAA–TOPO-capped CdSe QDs were exposed to air 30 min before MAA coating, a marked dose-dependent decrease in cell viability was observed, from 98 to 21%, at 62.5 μg/mL. Likewise, MAA–TOPO-capped CdSe QDs exposed to ultraviolet (UV) light (15 mW/cm^2^) showed a dramatic dose-dependent decrease in cell viability, with longer exposure times increasing toxicity (1–8 hr: 91% decrease in cell viability). It was concluded that prolonged exposure of QDs to oxidative and photolytic environments can cause decomposition of MAA–TOPO-capped CdSe QD nanocrystals. Relatively high concentrations of free Cd were observed in the medium of QD solutions exposed to air (126 ppm) and UV (82 ppm), with 6 ppm nonoxidized QD core material (CdSe) remaining in solution. QDs were also observed to decompose in 1 mM hydrogen peroxide, releasing free Cd ions (24 ppm). Derfus (2004) concluded that QD toxicity was relative to environmental conditions; CdSe QD-induced toxicity was observed only above concentrations exceeding 0.25 mg/mL and 1 hr of UV exposure. Adding one or two monolayers of ZnS to the QDs virtually eliminated cytotoxicity due to oxidation (using the same protocol). Although ZnS capping material significantly reduced ambient air oxidation, it did not fully eliminate photooxidation, with high levels of free Cd observed in solution after 8 hr under photooxidative conditions. BSA-coated ZnS-capped QDs were also found to have reduced cytotoxicity compared with non-BSA-coated ZnS-capped QDs at the same concentration (0.25 mg/mL). Aldana et al. (2001) also observed photochemical instability in thiol-coated CdSe QDs, although not at relevant UV wavelengths (254 nm). It was noted, however, that the photochemical stability of CdSe nanocrystals was closely related to the thickness and packing of the ligand monolayer.

Last, *Staphylococcus aureus* cultures exposed to transferrin-conjugated QDs showed marked increase in fluorescence after 2 weeks of exposure, attributed to intracellular oxidation of the QDs, with a marked increase in intracellular Se concentration. Kloepfer et al. (2003) observed the internalization of both free Cd and Se in *S. aureus* cells but not internalization of measurable transferrin-conjugated QDs. The authors also noted that photostability of the QD conjugates was an issue during preparation, and QD conjugation procedures were performed under little or no light to minimize QD photolysis.

### Intracellular and in vivo degradation.

Given that studies indicate QDs may be susceptible to photolysis and oxidation, the question arises as to their *in vivo*/intracellular oxidative stability, and a few studies suggest the possibility of intracellular degradation. Although Hoshino et al. (2004a) noted that CdSe/ZnS–SSA QDs could be observed in EL-4 cells for more than a week, with approximately 10% of the cells retaining QDs after 10 days in culture, the fluorescent intensity of cells was observed to gradually decrease and was highly concentrated in endosomes. QD fluorescence was possibly diminished by low pH, oxidation of QD surface structures, or intracellular factors adsorbed onto QD surfaces. Similarly, a substantial loss of QD fluorescence over time was noted by Gao et al. (2004) and Akerman et al. (2002) upon administration of QDs to live animals. Although the exact origin of the loss of QD fluorescence was not clear, Gao et al. (2004) stated that recent research in their group suggested that QD surface ligands and coatings were slowly degraded *in vivo*, leading to surface defects and fluorescence quenching. They noted, however, that QDs coated with a high-molecular-weight (100 kDa) copolymer and a grafted 8-carbon alkyl side chain demonstrated greater *in vivo* stability than those with simple polymer and amphiphilic lipid coatings. Similarly, Chen and Gerion (2004) attributed the lack of observable genotoxicity of QDs to a silica coating, which successfully prevented the interaction of Cd, Se, Zn, and sulfur with proteins and DNA in the nucleus.

### Cytotoxicity of QD capping materials.

Relative to *in vivo* degradation, Hoshino et al. (2004b) observed that QD surface coatings such as MUA may be detached under acidic and oxidative conditions in endosomes and released into cytoplasm. To assess the toxicity of surface coatings, Hoshino et al. (2004b) assayed three QD coating materials (MUA, cysteamine, and thioglycerol) and two possible impurities (TOPO and ZnS) for cytotoxicity. Treatment of WTK1 cells with MUA alone for 12 hr resulted in cytotoxicity at doses > 100 μg/mL. DNA damage was observed at 50 μg/mL (2 hr of treatment). Cysteamine was observed to be weakly genotoxic when cells were treated for 12 hr. The toxicity of thioglycerol was negligible. Hoshino et al. (2004b), observing TPOP to be cytotoxic and genotoxic, stated that removal of TPOP from the QD samples is important in reducing toxicity. Their findings provided evidence that QD-induced genotoxicity was not caused by the QD core but by hydrophilic QD coatings.

### Summary of QD toxicity.

The studies reviewed here suggest that QD toxicity depends on multiple factors derived from both the inherent physicochemical properties of QDs and environmental conditions. QD size, charge, concentration, outer coating bioactivity (capping material and functional groups), and oxidative, photolytic, and mechanical stability are each factors that, collectively and individually, can determine QD toxicity. Of these physicochemical characteristics, functional coating and QD core stability figure prominently and likely will be significant factors in assessing the risk of QD toxicity in real-world exposure scenarios.

## Absorption, Distribution, Metabolism, and Excretion of Quantum Dots *in Vivo*

Several studies have shown QDs may be systemically distributed and may accumulate in organs and tissues. Absorption, distribution, metabolism, and excretion (ADME) characteristics are, not surprisingly, highly variable for QDs because of the wide variation in QD physicochemical properties. QD size, charge, concentration, stability, and outer coating bioactivity each contribute to not only the potential toxicity of a given QD but also to their ADME characteristics. Physicochemical properties in conjunction with environmental factors and QD stability (oxidative and photolytic lability) together are a paradigm in which ADME characteristics of QDs can be highly variable and difficult to predict.

Several *in vitro* studies have shown QDs to be incorporated via endocytic mechanisms by a variety of cell types. Mammalian (HeLa) and *Dictyostelium discoideum* (AXS) cells were observed to incorporate avidin and DHLA-conjugated CdSe/ZnS QDs via endocytosis (Jaiswal et al. 2003), and rat primary hepatocytes were observed to incorporate CdSe–MAA QDs (Derfus 2004). Hoshino et al. (2004a) observed adherence of CdSe/ZnS–SSA QDs to the surface of EL-4 cells, with subsequent endocytosis and increase in cytosolic QD concentration in a time-dependent manner (minutes to hours). Other studies have shown similar nonspecific uptake. Hanaki et al. (2003), exposing Vero cells to CdSe/ZnS–MUA QDs coated with SSA, observed endosomal/lysosomal localization of the QDs near the perinuclear region 5 days after exposure. Parak et al. (2002) observed endocytosis and vesicular storage and transport of CdSe/ZnS silicon dioxide–coated QDs to the perinuclear region in human mammary tumor cells, and an *in vivo* study by Dubertret et al. (2002) demonstrated endocytosis and active transport of QD micelles (phospholipid block-copolymer) in *Xenopus* embryos.

In one instance, QD size was shown to be a determining factor in subcellular distribution. Lovric et al. (2005) observed 5.2 nm cationic CdTe QDs to localize throughout the cytoplasm of N9 cells (murine microglial cell line) but not in the nucleus. In contrast, 2.2-nm cationic CdTe QDs were observed to localize in the nuclear compartment within the same time frame. Hence, in this instance, size, not charge, was a determining factor in subcellular localization. It was noted, however, that because relatively unrestrained passage of macromolecules up to 9 nm in diameter occurs through nuclear pores, the size of the QDs (2.2 and 5.2 nm) cannot be the only explanation for the entry of smaller QDs (2.2 nm) into the nucleus. Altering the bioactivity of the smaller 2.2 nm CdTe QDs by conjugation to BSA was seen to limit its localization to the cytosol.

Where nonspecific endocytic mechanisms have been shown to be instrumental in QD uptake by cells, receptor-mediated processes may also contribute to cellular internalization when QDs carry bioactive moieties specific for cell receptor types or surface proteins. Epidermal growth factor (EGF)–conjugated CdSe/ZnS QDs proved to be highly specific for the EGF receptor (erbB1), demonstrating rapid internalization into endosomes of Chinese hamster ovary cells. The endocytic vesicles were observed to undergo a directed linear motion mediated by microtubule-associated motor proteins and vesicular fusion (Lidke et al. 2004). QDs coated with anti-Pgp showed good specificity for live HeLa cells transfected with Pgp-EGFP (EGF protein), with no apparent nonspecific cell labeling (Jaiswal et al. 2003). Other studies yielded similar results: CdSe/ZnS QDs conjugated to peptides specific for lung, vascular, and lymphatic tissues exhibited specificity for labeling cell membranes of their targeted tissue types (Akerman et al. 2002). Dahan et al. (2003) found that QDs conjugated with glycine receptor (GlyR1) ligands exhibited specificity for endogenous GlyR1 subunits on cultured spinal neurons. Last, prostate-specific membrane antigen–conjugated QDs specifically targeted prostate tumors in mice (Gao et al. 2004), and QDs complexed with a viral (SV40) nuclear localization signal peptide were observed to readily enter the nuclear compartment of human HeLa cells (Chen and Gerion 2004).

In invertebrate cell types, Kloepfer et al. (2003) observed transferrin-conjugated CdSe QDs to enter *S. aureus* bacterial cells, which do not endocytose but rely on membrane transporters. The transferrin-conjugated QDs also showed clear internal labeling in the fungi *Schizosacharomyces pombe* and *Penicillium chrysogenum*. No internal labeling of nonpathogenic staphylococci and micrococci was observed, and it was suggested that transferrin-mediated transport processes were involved in cell-specific uptake.

Importantly, where endocytic mechanisms have been observed in a variety of cell types, the question of systemic distribution arises. Although few *in vivo* studies exist, they suggest that QDs may be systemically distributed in rodent animal models and accumulate in a variety of organs and tissues. EL-4 cells containing CdSe/ZnS–SSA QDs (via endocytosis) were observed in the kidneys, liver, lung, and spleen of mice up to 7 days after injection, with spleen and lung having the most accumulation (fluorescence) (Hoshino et al. 2004a). Similarly, Ballou et al. (2004), employing QD coatings of different molecular weights [MW; methoxy-terminated PEG, MW 750 (mPEG-750), carboxy-terminated PEG, MW 3,400 (COOH–PEG-3400), and ethoxy-terminated PEG, MW 5,000 (mPEG-5000)], observed differential tissue and organ deposition in mice in a time- and size (MW)-dependent manner. For instance, mPEG-750 QDs and COOH–PEG-3400 QDs were cleared from circulation by 1 hr after injection, whereas mPEG-5000 QDs remained in circulation for at least 3 hr. At 24 hr after injection, mPEG-750 QDs were observed in the lymph nodes, liver, and bone marrow. In contrast, significantly less retention of COOH–PEG-3400 and mPEG-5000 QDs was observed in lymphatic tissue compared with bone marrow, liver, and spleen. At 133 days, continued fluorescence of mPEG-750 QDs was observed in the lymph nodes and bone marrow. A study by Akerman et al. (2002) yielded comparable findings. Lung- and tumor-targeting peptide-coated CdSe/ZnS QDs injected into mice (iv), regardless of the peptide used for the coating, accumulated in both the liver and spleen in addition to the targeted respiratory tissues. Interestingly, additionally coating CdSe/ZnS QDs with PEG (a polymer known to minimize molecular interactions and improve colloidal solubilities) nearly eliminated the nonspecific uptake of QDs into the liver and spleen. An *in vivo* study employing *Xenopus* embryos revealed that QDs, once internalized by cells, subsequently may be transferred to daughter cells on cell division. Dubertret et al. (2002), injecting a CdSe/ZnS micelle (PEG–PE and phosphatidylcholine) conjugated with an oligonucleotide into *Xenopus* embryos, observed QD labeling of all embryonic cell types, including somites, neurons, axonal tracks, ectoderm, neural crest, and endoderm. The internalized QDs were localized to both the cytosol and nuclear envelope and were transferred to daughter cells on cell division. The progeny of the QD-injected cells were shown to contain fluorescent QDs after several days of development.

Metabolic processes and excretory mechanisms involved in the elimination of QDs, as well as *in vivo* bioactivity, remain poorly understood and have not been well studied. *In vivo* studies suggest that, regardless of the specificity of the QD, vertebrate systems tend to recognize QDs as foreign, with elimination of the materials through the primary excretory organs/systems: the liver, spleen, and lymphatic systems. However, this a rough generalization, and discrepancies in the literature exist. For instance, subcutaneous injection of CdSe/ZnS–PEG-coated QDs in mice showed clearance of the QDs from the site of injection, with accumulation of QDs in lymph nodes. In contrast, Akerman et al. (2002) observed that modification of lung-and tumor-targeting peptide-conjugated CdSe/ZnS QDs with a PEG coating nearly eliminated nonspecific elimination of QDs via the lymphatic system.

The above studies suggest that QDs may see variable systemic distribution dependent on individual QD physicochemical properties. Although studies are limited, QD tissue/organ distribution seems to be multifactorial, depending on QD size, QD core–shell components, and the bioactivity of conjugated or otherwise attached functional groups. Size alone can markedly affect distribution kinetics, and QD surface coating can govern serum lifetime and pattern of deposition (Ballou et al. 2004; Lovric et al. 2005). QDs lacking specialized functional groups or specificity have been shown to be incorporated via endocytic mechanisms by a variety of cell types, both *in vivo* and *in vitro*. In contrast, QDs bearing natural ligands specific for cell receptors and cell membrane proteins have been shown to be specific for given cell membrane proteins/receptor types. Several studies have shown nonspecific QDs to adhere to cell surfaces, possibly through interactions of QD with glycoproteins and glycoplipids in cell membranes. Although many studies indicate endocytic processes and intracellular vesicular trafficking and storage of QDs, the exact mechanisms remain to be elucidated. Subcellular localization is variable, like systemic distribution, and dependent on QD physicochemical properties. Such variables, determined by the unique physicochemical properties of individual QD types, will prove significant in developing characterization protocols for QD toxicity screening, given the nonuniformity in size, QD functional coatings, core–shell complexes, and outer coating photolytic and oxidative stability.

## Correlation of Quantum Dot Concentrations and Toxicity

Quantum dot dosage/exposure concentrations reported in the literature vary widely in units of measurement (e.g., micrograms per milliliter, molarity, milligrams per kilogram body weight, QDs per cell), and correlating dosage across studies is currently challenging. Further, some QDs were found to be cytotoxic only after degradation of their core coatings both *in vivo* and/or *in vitro*. Nevertheless, reported values of dose–response relationships can be assessed in Table 1. Of note, those studies that observed no cytotoxicity generally employed protocols that used short-term acute exposures, where cells were in contact with QDs for 15 min to 8 hr (e.g., Hanaki et al. 2003; Jaiswal et al. 2003; Voura et al. 2004). For instance, studies by Jaiswal et al. (2003), in which no cytotoxicity was observed, employed acute exposures of cells to QDs for 15 min to 2 hr, after which time cells where washed and observations made. Similar exposure times (2 hr) were employed by Hanaki et al. (2003). In contrast, QD-induced cytotoxicity was generally found in those studies that tended to be longer in nature, with exposure times from 2 hr to several days. For example, Hoshino et al. (2004a), Shiohara et al. (2004), and Lovric et al. (2005) employed 24-hr exposures.

## Discussion

Cadmium and selenium, two of the most widely used constituent metals in QD core metalloid complexes, are known to cause acute and chronic toxicities in vertebrates and are of considerable human health and environmental concern (Fan et al. 2002; Hamilton 2004; Henson and Chedrese 2004; Kondoh et al. 2002; Poliandri et al. 2003; Satarug and Moore 2004; Spallholz and Hoffman 2002). For instance, Cd, a probable carcinogen, has a biologic half-life of 15–20 years in humans, bioaccumulates, can cross the blood–brain barrier and placenta, and is systemically distributed to all bodily tissues, with liver and kidney being target organs of toxicity. The potential environmental impacts of Se contamination are well understood from Kesterson Reservoir, California, and Belews Lake, North Carolina, where a marked impact on the local ecosystem resulted from elevated environmental concentrations of Se. Because of QD metalloid core composition, the uniqueness of each type of QD, the oxidative and photochemical lability of certain types of QDs, and the dearth of information on routes of exposure and the environmental transport and fate of QD materials, the potential risks posed by QD materials to human health and the environment should be seriously considered.

The likely increase in prevalence of QD products in society, in tandem with their potential toxicity, necessitates elucidation of the potential adverse effects of these materials, not only for the protection of human health and environmental integrity but also to aid industry and regulatory bodies in maximizing the use of these materials. Given the potential societal benefits offered by QD technology, elucidating the mechanisms and sources of QD toxicity will help avoid the pitfalls encountered by the misapplication of previous technologies. Nanotoxicologic information, currently lacking, will be vital to this process, in aiding industry in producing QDs of minimal risk, and in elucidating the mechanisms of action of QDs, as well as their environmental transport and fate. Only with this knowledge can the biocompatibility of QD technology with the social and ecologic systems in which these materials will be applied be achieved, and can we ensure that this technology develops responsibly, with sound public support.

## Summary

The studies reviewed here suggest several key points, in particular, that not all QDs are alike and that engineered QDs cannot be considered a uniform group of nanomaterials. QD ADME and toxicity depend on multiple factors derived from both inherent physicochemical properties and environmental conditions; QD size, charge, concentration, outer coating bioactivity (capping material and functional groups), and oxidative, photolytic, and mechanical stability have each been implicated as determining factors in QD toxicity. Hence, it is likely that grouping or classification of QDs as to their potential toxicities based on size or other physicochemical properties alone will, early on, prove troublesome, and each QD type will need to be characterized individually as to its potential toxicity. In summary, the findings in these reviews suggest that under certain conditions QDs may pose environmental and human health risks as determined by rodent animal models and *in vitro* cell cultures.

## Figures and Tables

**Table 1:** Review articles summary of QD types, exposure concentrations, experimental conditions, and observed toxicity.

QD	Model	Exposure conditions/administration	QD concentration	Exposure duration	Toxicity	Reference
CdSe/ZnS–SSA	EL-4 cells	1 × 10^6^ cells/well	0.1–0.4 mg/mL	0–24 hr	Cytotoxic: 0.1 mg/mL altered cell growth; most cells nonviable at 0.4 mg/mL	Hoshino et al. 2004a
CdSe/ZnS–SSA	EL-4 cells	200 μL cell suspension injected (iv) into mice	0.1 mg/mL QDs per 5 × 10^7^ cells	2 hr to 7 days	No toxicity in mice *in vivo*	Hoshino et al. 2004a
CdSe/ZnS conjugates: NH_2_, OH, OH/COOH, H_2_/OH, MUA, COOH	WTK1 cells	5 × 10^4^ cells/mL	1–2 μM	12 hr	2 μM QD–COOH induced DNA damage at 2 hr DNA repair on prolonged incubation (12 hr)	Hoshino et al. 2004b
CdSe/ZnS–MUA	Vero, HeLa, and primary human hepatocytes	100 μL QDs/3 × 10^4^ cells	0–0.4 mg/mL	24 hr	Cytotoxic: 0.2 mg/mL, Vero; 0.1 mg/mL, HeLa; 0.1 mg/mL, hepatocytes;	Shiohara et al. 2004
CdTe	Rat pheochromocytoma cells, murine microglial cells	1 × 10^5^ cells/cm^2^	0.01–100 μg/mL	2–24 hr	10 μg/mL cytotoxic	Lovric et al. 2005
CdSe–MAA, TOPO QDs	Primary rat hepatocytes		62.5–1,000 μg/mL	1–8 hr	Cytotoxic: 62.5 μg/mL cytotoxic under oxidative/photolytic conditions No toxicity on addition of ZnS cap	Derfus 2004
QD micelles: CdSe/ZnS QDs in (PEG–PE) and phosphatydilcholine	*Xenopus* blastomeres	5 × 10^9^ QDs/cell (~ 0.23 pmol/cell)	1.5–3 nL of 2.3 μM QDs injected, ~ 2.1 × 10^9^ to 4.2 × 10^9^ injected QDs/cell	Days	5 × 10^9^ QDs/cell: cell abnormalities, altered viability and motility No toxicity at 2 × 10^9^ QDs/cell	Dubertret et al. 2002
CdSe/ZnS amp-QDs, and mPEG QDs	Mice	200-μL tail vein injection	Injections; ~ 180 nM QD, ~ 20 pmol QD/g animal weight	15 min cell incubations, 1–133 days *in vivo*	No signs of localized necrosis at the sites of deposition	Ballou et al. 2004
CdSe/ZnS–DHLA	*Dictyostelium discoideum* and HeLa cells		400–600 nM	45–60 min	No effects on cell growth	Jaiswal et al. 2003
Avidin-conjugated CdSe/ZnS QDs	HeLa cells		0.5–1.0 μM	15 min	No effect on cell growth, development	Jaiswal et al. 2003
CdSe/ZnS–amphiphilic micelle	Mice	Tail vein injection	60 μM QD/g animal weight, 1 μM and 20 nM final QD concentration	Not given	Mice showed no noticeable ill effects after imaging	Larson et al. 2003
CdSe/ZnS–DHLA QDs	Mice, B16F10 cells	5 × 10^4^ B16F10 cells with 10 μL QDs (~ 10 pmol), tail vein (iv) injection	100 μL of B16F10 cells used for tail vein injection, ~ 2 × 10^5^ to 4 × 10^5^ cells injected	4–6 hr cell incubation, mice sacrificed at 1–6 hr	No toxicity observed in cells or mice	Voura et al. 2004
CdSe/ZnS–MUA QDs; QD–SSA complexes	Vero cells	0.4 mg/mL	0.24 mg/mL	2 hr	0.4 mg/mL MUA/SSA–QD complexes did not affect viability of Vero cells	Hanaki et al. 2003
CdSe/ZnS	HeLa cells	1 × 10^6^ cells	10 pmol QDs/1 × 10^5^ cells (~ 10 nM)	10 days (cell culture)	10 nM QD had minimal impact on cell survival	Chen and Gerion 2004
